# Supplementary material for: A Likelihood Approach for Real-Time Calibration of Stochastic Compartmental Epidemic Models
Source: PLoS Comput Biol. 2017 Jan 17;13(1):e1005257. doi: 10.1371/journal.pcbi.1005257 (PMC5240920; doi:10.1371/journal.pcbi.1005257)
Supplement: S1 File — (TAR.GZ) [file pcbi.1005257.s014.tar.gz › HSPH_Online-SI-Revision/IPoi09/n1-severe/n1-severe_BENCH2_table4.pdf]

1	newly 3 weeks spec	24.	26.4615	{12., 47.}	47.9487	0.193788	newly 3 weeks cumul	81.	94.2769	{48., 163.}	45.2612	0.177086	newly 5 weeks spec	28.	17.9385	{6., 37.}	52.1978	0.338654	newly 5 weeks cumul	135.	134.062	{64., 220.}	40.3761	0.183958	newly 50 weeks cumul	286.	209.923	{93., 354.}	45.2448	0.261592
2	newly 3 weeks spec	23.	24.2466	{8., 47.}	55.2114	0.253746	newly 3 weeks cumul	90.	86.9452	{36., 157.}	44.4597	0.214226	newly 5 weeks spec	19.	15.2877	{5., 31.}	54.7224	0.31266	newly 5 weeks cumul	138.	121.74	{45., 228.}	46.2478	0.240503	newly 52 weeks cumul	204.	184.041	{64., 398.}	51.3497	0.259109
3	newly 3 weeks spec	38.	38.3971	{17., 63.}	38.8158	0.20909	newly 3 weeks cumul	102.	125.015	{72., 188.}	40.4412	0.173794	newly 5 weeks spec	22.	26.6176	{11., 52.}	56.4171	0.237327	newly 5 weeks cumul	152.	183.191	{99., 290.}	42.7148	0.186813	newly 51 weeks cumul	261.	308.044	{136., 603.}	51.403	0.222458
4	newly 3 weeks spec	24.	41.7424	{23., 70.}	78.0934	0.226471	newly 3 weeks cumul	94.	150.833	{91., 218.}	62.3952	0.193039	newly 5 weeks spec	9.	27.1212	{12., 46.}	202.02	0.415958	newly 5 weeks cumul	116.	211.621	{123., 324.}	83.0329	0.237406	newly 54 weeks cumul	163.	309.333	{159., 481.}	90.9091	0.24323
5	newly 3 weeks spec	23.	32.7778	{17., 50.}	55.7971	0.183116	newly 3 weeks cumul	56.	127.236	{74., 185.}	127.207	0.328148	newly 5 weeks spec	17.	19.8333	{10., 33.}	41.1765	0.158983	newly 5 weeks cumul	100.	172.319	{97., 254.}	74.9583	0.21914	newly 53 weeks cumul	148.	243.264	{133., 381.}	69.7729	0.207835
6	newly 3 weeks spec	43.	34.4821	{17., 56.}	33.8455	0.191409	newly 3 weeks cumul	120.	119.964	{77., 175.}	28.0357	0.132647	newly 5 weeks spec	35.	26.1429	{12., 46.}	40.102	0.243544	newly 5 weeks cumul	192.	176.25	{104., 271.}	28.8132	0.14761	newly 50 weeks cumul	340.	320.982	{158., 534.}	37.0641	0.175
7	newly 3 weeks spec	17.	21.5522	{9., 39.}	57.5066	0.210003	newly 3 weeks cumul	65.	76.4925	{39., 134.}	44.4546	0.172298	newly 5 weeks spec	9.	14.0746	{4., 28.}	81.2604	0.267362	newly 5 weeks cumul	90.	108.493	{53., 198.}	47.0149	0.177265	newly 50 weeks cumul	125.	163.821	{70., 324.}	56.0597	0.195468
8	newly 3 weeks spec	6.	33.0143	{17., 51.}	450.238	0.694903	newly 3 weeks cumul	53.	127.2	{73., 175.}	140.916	0.355788	newly 5 weeks spec	0.1	19.4714	{9., 32.}	19 371.4	2.24203	newly 5 weeks cumul	55.	171.757	{97., 237.}	212.286	0.464319	newly 54 weeks cumul	55.	228.214	{125., 323.}	314.935	0.58162
9	newly 3 weeks spec	21.	31.0339	{13., 54.}	68.2809	0.22548	newly 3 weeks cumul	83.	106.458	{50., 167.}	45.8648	0.167346	newly 5 weeks spec	22.	22.4407	{7., 43.}	47.3035	0.230311	newly 5 weeks cumul	119.	156.831	{67., 274.}	50.6481	0.181608	newly 50 weeks cumul	236.	260.424	{109., 503.}	45.7986	0.192601
10	newly 3 weeks spec	16.	31.7167	{11., 58.}	109.063	0.285014	newly 3 weeks cumul	74.	109.35	{42., 176.}	66.509	0.214391	newly 5 weeks spec	16.	21.8333	{6., 41.}	74.375	0.259392	newly 5 weeks cumul	103.	157.817	{59., 270.}	70.8252	0.218718	newly 50 weeks cumul	137.	248.083	{84., 434.}	96.4112	0.253796
11	newly 3 weeks spec	26.	26.6316	{11., 48.}	44.5344	0.203093	newly 3 weeks cumul	70.	89.4386	{49., 144.}	44.8622	0.158922	newly 5 weeks spec	28.	21.4211	{7., 43.}	49.1855	0.310487	newly 5 weeks cumul	115.	135.053	{67., 234.}	45.3852	0.175842	newly 47 weeks cumul	274.	239.123	{111., 443.}	44.103	0.230373
12	newly 3 weeks spec	14.	20.8784	{8., 39.}	69.9807	0.215584	newly 3 weeks cumul	54.	87.4459	{40., 158.}	72.8979	0.210382	newly 5 weeks spec	7.	10.4189	{4., 19.}	75.8687	0.241643	newly 5 weeks cumul	71.	111.878	{51., 205.}	70.4416	0.206531	newly 55 weeks cumul	74.	134.189	{61., 237.}	89.1161	0.236944
13	newly 3 weeks spec	28.	28.2985	{11., 50.}	45.2026	0.234732	newly 3 weeks cumul	97.	99.7164	{49., 174.}	37.6058	0.204059	newly 5 weeks spec	15.	19.4925	{6., 35.}	62.3881	0.254572	newly 5 weeks cumul	134.	142.358	{65., 249.}	40.9223	0.214571	newly 50 weeks cumul	214.	219.985	{86., 407.}	43.709	0.231385
14	newly 3 weeks spec	29.	39.7432	{26., 58.}	44.0354	0.145652	newly 3 weeks cumul	89.	153.378	{101., 228.}	73.5196	0.220625	newly 5 weeks spec	15.	23.9865	{12., 38.}	68.3784	0.20126	newly 5 weeks cumul	126.	208.811	{136., 306.}	67.8893	0.205181	newly 55 weeks cumul	170.	282.878	{168., 406.}	69.2607	0.203815
15	newly 3 weeks spec	13.	15.3099	{5., 31.}	61.1051	0.248032	newly 3 weeks cumul	54.	58.7324	{21., 113.}	53.9384	0.230917	newly 5 weeks spec	3.	9.71831	{3., 24.}	230.516	0.430216	newly 5 weeks cumul	64.	81.4366	{29., 164.}	61.4877	0.222459	newly 49 weeks cumul	74.	119.465	{40., 270.}	84.9258	0.246346
16	newly 3 weeks spec	18.	34.4143	{15., 54.}	97.381	0.265677	newly 3 weeks cumul	97.	130.2	{67., 208.}	46.7452	0.160895	newly 5 weeks spec	13.	20.	{9., 33.}	68.5714	0.209567	newly 5 weeks cumul	125.	176.057	{95., 294.}	52.2286	0.171648	newly 55 weeks cumul	201.	235.986	{127., 360.}	39.1542	0.150062
17	newly 3 weeks spec	13.	24.9286	{14., 38.}	94.6154	0.2598	newly 3 weeks cumul	54.	106.8	{67., 154.}	98.9418	0.275375	newly 5 weeks spec	4.	14.0857	{6., 21.}	252.143	0.488871	newly 5 weeks cumul	73.	139.314	{88., 202.}	92.0157	0.258666	newly 55 weeks cumul	87.	176.6	{107., 266.}	104.335	0.284319
18	newly 3 weeks spec	19.	34.2909	{10., 59.}	99.2344	0.298541	newly 3 weeks cumul	61.	127.709	{45., 200.}	119.613	0.324977	newly 5 weeks spec	28.	22.7818	{4., 48.}	55.3896	0.389951	newly 5 weeks cumul	119.	178.509	{66., 281.}	69.9007	0.239204	newly 55 weeks cumul	388.	252.836	{85., 440.}	45.4358	0.306416
19	newly 3 weeks spec	15.	21.8214	{8., 43.}	70.7143	0.230297	newly 3 weeks cumul	71.	78.4286	{31., 147.}	43.3099	0.178233	newly 5 weeks spec	12.	15.5714	{4., 35.}	72.0238	0.265858	newly 5 weeks cumul	93.	112.732	{47., 229.}	49.2127	0.180558	newly 48 weeks cumul	186.	176.964	{70., 362.}	50.4416	0.22874
20	newly 3 weeks spec	7.	19.8923	{7., 37.}	187.253	0.391743	newly 3 weeks cumul	54.	75.3385	{31., 126.}	58.7179	0.197963	newly 5 weeks spec	3.	13.5846	{5., 26.}	353.846	0.566298	newly 5 weeks cumul	64.	106.015	{43., 193.}	78.3894	0.229217	newly 48 weeks cumul	97.	159.215	{65., 324.}	78.9215	0.227628
21	newly 3 weeks spec	33.	16.2727	{6., 29.}	56.933	0.426593	newly 3 weeks cumul	107.	69.8182	{33., 127.}	45.5395	0.286972	newly 5 weeks spec	12.	8.45455	{2., 18.}	52.0202	0.373387	newly 5 weeks cumul	135.	90.0758	{41., 171.}	45.8025	0.28457	newly 55 weeks cumul	223.	110.53	{49., 201.}	54.1582	0.387237
22	newly 3 weeks spec	12.	26.6667	{9., 53.}	131.579	0.31664	newly 3 weeks cumul	57.	93.	{33., 166.}	79.84	0.236358	newly 5 weeks spec	7.	16.9649	{5., 33.}	157.393	0.340429	newly 5 weeks cumul	75.	131.702	{48., 247.}	91.2281	0.251918	newly 49 weeks cumul	81.	205.421	{62., 381.}	160.191	0.334441
23	newly 3 weeks spec	21.	24.8361	{8., 46.}	54.9571	0.21814	newly 3 weeks cumul	88.	92.4262	{42., 165.}	46.386	0.202387	newly 5 weeks spec	16.	18.541	{4., 40.}	74.8975	0.324415	newly 5 weeks cumul	122.	131.967	{59., 243.}	51.7334	0.221189	newly 51 weeks cumul	188.	199.623	{76., 383.}	58.5542	0.252552
24	newly 3 weeks spec	15.	13.7344	{4., 27.}	51.7708	0.289619	newly 3 weeks cumul	68.	52.5	{22., 97.}	46.2776	0.268522	newly 5 weeks spec	6.	8.45313	{2., 15.}	82.0313	0.302606	newly 5 weeks cumul	79.	71.7813	{29., 148.}	48.4573	0.243148	newly 48 weeks cumul	103.	103.234	{40., 204.}	53.747	0.247297
25	newly 3 weeks spec	9.	18.6308	{6., 38.}	121.026	0.308778	newly 3 weeks cumul	59.	68.2769	{28., 127.}	49.2047	0.201677	newly 5 weeks spec	11.	14.9231	{5., 29.}	77.0629	0.252071	newly 5 weeks cumul	83.	98.7538	{41., 175.}	52.9008	0.203786	newly 46 weeks cumul	100.	177.369	{60., 305.}	94.9077	0.242878
26	newly 3 weeks spec	16.	20.4444	{9., 36.}	51.5625	0.180849	newly 3 weeks cumul	56.	79.4028	{42., 133.}	55.0843	0.17908	newly 5 weeks spec	15.	11.75	{4., 20.}	44.8148	0.249258	newly 5 weeks cumul	84.	106.208	{54., 173.}	46.0813	0.162715	newly 53 weeks cumul	169.	144.25	{71., 235.}	37.7055	0.196071
27	newly 3 weeks spec	8.	34.5238	{14., 60.}	331.548	0.564995	newly 3 weeks cumul	28.	122.873	{57., 195.}	338.832	0.597389	newly 5 weeks spec	0.1	22.8413	{9., 39.}	22 741.3	2.2765	newly 5 weeks cumul	28.	174.302	{82., 288.}	522.506	0.744306	newly 52 weeks cumul	28.	258.079	{115., 439.}	821.712	0.898432
28	newly 3 weeks spec	36.	34.9194	{16., 61.}	38.3961	0.18417	newly 3 weeks cumul	124.	133.145	{80., 204.}	31.5687	0.129968	newly 5 weeks spec	31.	22.0323	{11., 42.}	44.1207	0.260303	newly 5 weeks cumul	183.	183.774	{101., 314.}	31.9408	0.139658	newly 55 weeks cumul	256.	250.71	{142., 455.}	34.06	0.153839
29	newly 3 weeks spec	15.	15.1563	{2., 32.}	63.9583	0.33116	newly 3 weeks cumul	52.	57.8594	{20., 113.}	54.4772	0.226665	newly 5 weeks spec	10.	9.73438	{3., 23.}	68.5938	0.342914	newly 5 weeks cumul	71.	80.1719	{27., 163.}	56.9762	0.228186	newly 48 weeks cumul	150.	123.5	{37., 260.}	58.0417	0.306848
30	newly 3 weeks spec	15.	20.6714	{4., 45.}	74.9524	0.276052	newly 3 weeks cumul	70.	74.9429	{25., 161.}	53.6735	0.236744	newly 5 weeks spec	2.	13.0857	{3., 27.}	555.714	0.679292	newly 5 weeks cumul	78.	104.2	{32., 224.}	66.6667	0.2328	newly 49 weeks cumul	112.	156.929	{48., 347.}	73.4566	0.24252
31	newly 3 weeks spec	19.	21.0154	{7., 42.}	52.2267	0.230319	newly 3 weeks cumul	55.	71.7385	{28., 117.}	51.9161	0.182974	newly 5 weeks spec	24.	14.1385	{4., 34.}	57.1154	0.401794	newly 5 weeks cumul	98.	103.385	{39., 182.}	43.7677	0.188974	newly 50 weeks cumul	218.	163.569	{63., 310.}	51.4185	0.290782
32	newly 3 weeks spec	37.	29.5686	{11., 51.}	37.9968	0.221991	newly 3 weeks cumul	98.	108.255	{52., 176.}	39.1156	0.165869	newly 5 weeks spec	27.	20.4902	{5., 41.}	49.6732	0.325709	newly 5 weeks cumul	151.	153.627	{67., 240.}	38.7482	0.181009	newly 52 weeks cumul	280.	233.49	{89., 414.}	43.3333	0.244118
33	newly 3 weeks spec	31.	35.0263	{19., 55.}	36.927	0.142021	newly 3 weeks cumul	119.	139.368	{83., 203.}	32.3972	0.123052	newly 5 weeks spec	13.	20.6447	{10., 35.}	70.9514	0.21535	newly 5 weeks cumul	150.	187.079	{110., 275.}	36.7018	0.130839	newly 55 weeks cumul	184.	246.25	{141., 402.}	43.8859	0.145726
34	newly 3 weeks spec	15.	10.3913	{3., 21.}	58.1643	0.365794	newly 3 weeks cumul	64.	39.1739	{15., 77.}	53.3741	0.346484	newly 5 weeks spec	10.	6.50725	{1., 13.}	59.5652	0.453446	newly 5 weeks cumul	87.	54.4493	{22., 110.}	53.8731	0.34167	newly 47 weeks cumul	179.	76.4203	{30., 142.}	62.7803	0.481993
35	newly 3 weeks spec	21.	34.7333	{17., 56.}	74.7937	0.221035	newly 3 weeks cumul	87.	141.107	{81., 218.}	65.5939	0.196056	newly 5 weeks spec	3.	19.72	{10., 37.}	557.333	0.762874	newly 5 weeks cumul	98.	187.36	{107., 285.}	91.7551	0.252241	newly 55 weeks cumul	98.	247.56	{139., 405.}	152.612	0.365103
36	newly 3 weeks spec	1.	9.35821	{2., 21.}	835.821	0.805994	newly 3 weeks cumul	11.	46.9403	{17., 88.}	327.001	0.541191	newly 5 weeks spec	0.1	4.16418	{1., 9.}	4073.13	1.44171	newly 5 weeks cumul	11.	57.0597	{21., 118.}	418.725	0.622238	newly 56 weeks cumul	11.	64.6716	{24., 128.}	487.924	0.671902
37	newly 3 weeks spec	19.	13.3279	{5., 24.}	45.384	0.284411	newly 3 weeks cumul	60.	53.1639	{24., 100.}	39.3716	0.210261	newly 5 weeks spec	11.	9.27869	{3., 18.}	46.6468	0.257947	newly 5 weeks cumul	82.	74.4262	{32., 139.}	40.1439	0.202901	newly 46 weeks cumul	171.	127.951	{48., 235.}	51.3469	0.288786
38	newly 3 weeks spec	5.	25.274	{10., 42.}	407.671	0.643332	newly 3 weeks cumul	41.	101.466	{47., 158.}	147.812	0.347566	newly 5 weeks spec	3.	14.6575	{6., 28.}	388.584	0.622554	newly 5 weeks cumul	46.	135.589	{66., 210.}	194.759	0.421051	newly 54 weeks cumul	51.	176.699	{84., 277.}	246.468	0.487363
39	newly 3 weeks spec	20.	29.4412	{13., 53.}	66.7647	0.243613	newly 3 weeks cumul	64.	105.029	{53., 170.}	72.8401	0.253344	newly 5 weeks spec	14.	21.2794	{8., 45.}	76.3655	0.260247	newly 5 weeks cumul	91.	150.471	{75., 278.}	74.8222	0.256597	newly 50 weeks cumul	151.	237.882	{109., 476.}	73.5294	0.257134
40	newly 3 weeks spec	21.	19.4	{4., 34.}	61.3919	0.321687	newly 3 weeks cumul	63.	85.2923	{28., 155.}	72.0147	0.248628	newly 5 weeks spec	9.	8.10769	{1., 17.}	73.8462	0.46066	newly 5 weeks cumul	89.	106.046	{30., 206.}	67.1737	0.263671	newly 56 weeks cumul	118.	122.431	{31., 236.}	63.4159	0.288202
41	newly 3 weeks spec	14.	30.3026	{14., 53.}	120.207	0.290306	newly 3 weeks cumul	52.	114.329	{61., 200.}	120.774	0.300044	newly 5 weeks spec	6.	16.9868	{7., 29.}	187.5	0.391052	newly 5 weeks cumul	63.	153.829	{81., 259.}	144.967	0.341837	newly 53 weeks cumul	66.	211.671	{106., 353.}	220.754	0.446744
42	newly 3 weeks spec	25.	20.9623	{9., 34.}	40.9057	0.213635	newly 3 weeks cumul	82.	81.0377	{42., 138.}	37.5288	0.171551	newly 5 weeks spec	15.	11.7736	{3., 24.}	55.9748	0.34477	newly 5 weeks cumul	118.	108.717	{56., 209.}	40.7739	0.195699	newly 53 weeks cumul	257.	141.604	{64., 278.}	52.5218	0.354414
43	newly 3 weeks spec	9.	19.9661	{8., 33.}	129.002	0.314644	newly 3 weeks cumul	28.	74.4237	{35., 133.}	168.341	0.384949	newly 5 weeks spec	9.	15.1356	{6., 29.}	88.8889	0.255154	newly 5 weeks cumul	44.	106.644	{48., 187.}	144.992	0.341006	newly 46 weeks cumul	120.	181.254	{70., 313.}	71.3559	0.214066
44	newly 3 weeks spec	16.	31.5714	{16., 51.}	100.731	0.271657	newly 3 weeks cumul	64.	123.39	{75., 185.}	94.2979	0.263169	newly 5 weeks spec	5.	19.2597	{9., 34.}	286.234	0.530319	newly 5 weeks cumul	85.	167.987	{101., 258.}	99.343	0.272105	newly 54 weeks cumul	109.	231.636	{133., 369.}	114.345	0.297767
45	newly 3 weeks spec	17.	27.3284	{14., 43.}	68.6567	0.202219	newly 3 weeks cumul	55.	103.657	{59., 167.}	90.8548	0.24943	newly 5 weeks spec	8.	16.9851	{7., 33.}	117.537	0.289125	newly 5 weeks cumul	80.	142.463	{75., 235.}	81.7351	0.228281	newly 51 weeks cumul	157.	200.791	{101., 325.}	48.0084	0.167589
46	newly 3 weeks spec	36.	29.3065	{10., 52.}	40.009	0.228903	newly 3 weeks cumul	87.	98.0806	{45., 148.}	38.3204	0.16411	newly 5 weeks spec	13.	20.6774	{7., 38.}	80.397	0.247007	newly 5 weeks cumul	135.	143.79	{64., 226.}	37.5508	0.16811	newly 50 weeks cumul	318.	238.855	{97., 401.}	44.2027	0.258946
47	newly 3 weeks spec	0.1	26.3939	{11., 46.}	26 293.9	2.34091	newly 3 weeks cumul	20.	111.318	{48., 194.}	456.591	0.682092	newly 5 weeks spec	0.1	13.3788	{4., 29.}	13 278.8	1.98701	newly 5 weeks cumul	20.	144.091	{64., 239.}	620.455	0.788672	newly 56 weeks cumul	20.	172.333	{77., 284.}	761.667	0.863008
48	newly 3 weeks spec	16.	18.	{6., 37.}	51.1538	0.219861	newly 3 weeks cumul	39.	70.4923	{30., 129.}	89.9014	0.242667	newly 5 weeks spec	12.	11.2923	{5., 22.}	51.5385	0.253418	newly 5 weeks cumul	57.	95.7077	{40., 164.}	80.108	0.225178	newly 50 weeks cumul	102.	135.615	{55., 245.}	57.632	0.193136
49	newly 3 weeks spec	13.	23.0986	{11., 43.}	86.7822	0.238499	newly 3 weeks cumul	64.	94.2535	{50., 171.}	56.6461	0.174322	newly 5 weeks spec	9.	13.493	{5., 23.}	69.6401	0.226198	newly 5 weeks cumul	90.	126.042	{66., 227.}	51.8466	0.16413	newly 54 weeks cumul	127.	164.451	{86., 298.}	46.3902	0.155767
50	newly 3 weeks spec	21.	34.2	{16., 52.}	71.746	0.223393	newly 3 weeks cumul	56.	122.35	{68., 175.}	120.387	0.320782	newly 5 weeks spec	17.	22.6167	{10., 39.}	52.2549	0.183102	newly 5 weeks cumul	91.	172.483	{93., 249.}	92.3993	0.261834	newly 52 weeks cumul	172.	263.217	{129., 365.}	63.9438	0.204912
